# Supplementary material for: Unite and Conquer: Association of Two G-Quadruplex Aptamers Provides Antiproliferative and Antimigration Activity for Cells from High-Grade Glioma Patients
Source: Pharmaceuticals (Basel). 2024 Oct 26;17(11):1435. doi: 10.3390/ph17111435 (PMC11597096; doi:10.3390/ph17111435)
Supplement: Supplementary file 1 [file pharmaceuticals-17-01435-s001.zip › pharmaceuticals-3246160-supplementary.pdf]

Supplementary Data

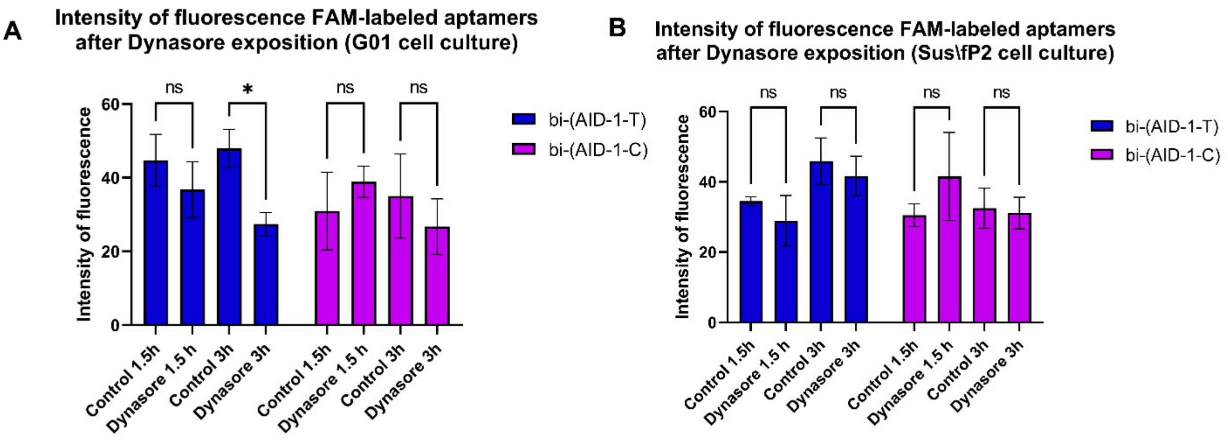

Figure S1. Fluorescence changes of FAM-labeled aptamers after Dynasore exposure

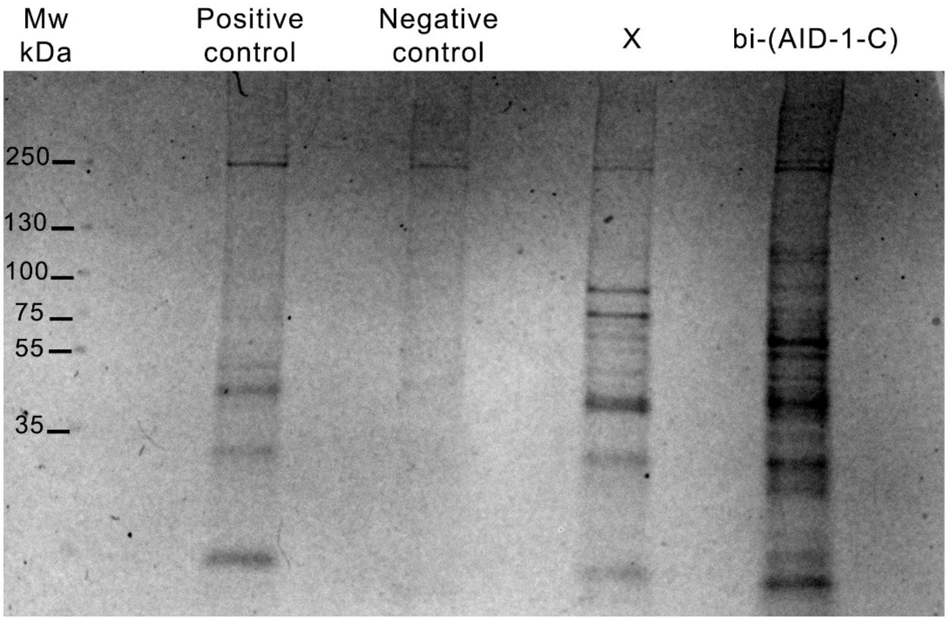

Figure S2. Full 4-20% PAGE electrophoresis of proteins obtained after incubation of cell lysates with biotin-labeled control oligonucleotide (positive control) and biotin-labeled bi-(AID-1-C) after the first column elution. The negative control contains oligonucleotide-untreated protein lysate after the first elution.

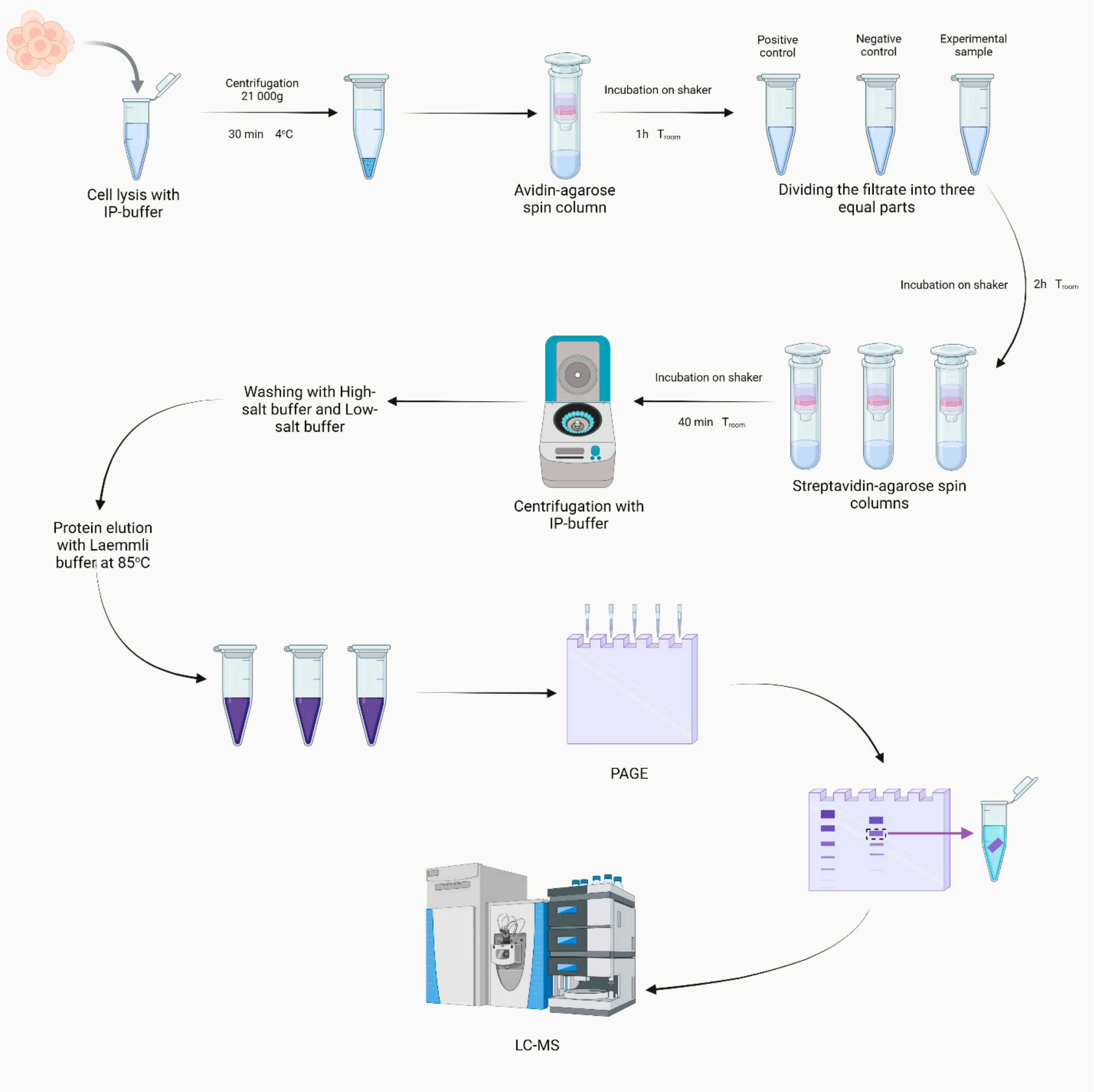

Figure S3. Assessment of aptamers specificity to the target protein. Created in BioRender. Pavlova, G. (2024) BioRender.com/t64u759

Table S1. Proteins specifically bound to biotin-labeled bi-(AID-1-T)

**Protein ID**	**Accession**	**-10lgP**	**Coverage (%)**	**Coverage (%) Sample 2**	**Area Sample 2**	**#Peptides**	**#Unique**	**#Spec Sample 2**	**PTM**			**Avg. Mass**	**Description**
P09874	PARP1_HUMAN	219.24	36	36	1.60E+08	60	60	114	Carbamidomethylation	Oxidation (M)	Acetylation (Protein N-term)	113084	Poly [ADP-ribose] polymerase 1 OS=Homo sapiens OX=9606 GN=PARP1 PE=1 SV=4
Q9H2U1	DHX36_H	211.11	28	28	1.44E+08	47	47	101	Carbamidomet	Oxidation (M)	Acetylation (Protein N-term)	114760	ATP-dependent DNA/RNA helicase DHX36 OS=Homo

	UMAN								hylation			sapiens OX=9606 GN=DHX36 PE=1 SV=2
Q1KMD3	HNRL2_HUMAN	166.63	27	27	7.70E+07	29	29	92	Carbamidomethylation	Oxidation (M)	85105	Heterogeneous nuclear ribonucleoprotein U-like protein 2 OS=Homo sapiens OX=9606 GN=HNRNPUL2 PE=1 SV=1
P68371	TBB4B_HUMAN	125.29	16	16	0	7	1	24	Carbamidomethylation	Oxidation (M)	49831	Tubulin beta-4B chain OS=Homo sapiens OX=9606 GN=TUBB4B PE=1 SV=1
Q07157	ZO1_HUMAN	119.68	7	7	2.47E+06	15	13	18			195457	Tight junction protein ZO-1 OS=Homo sapiens OX=9606 GN=TJP1 PE=1 SV=3
P16615	AT2A2_HUMAN	114.27	12	12	2.83E+06	14	14	16	Carbamidomethylation	Oxidation (M)	114757	Sarcoplasmic/endoplasmic reticulum calcium ATPase 2 OS=Homo sapiens OX=9606 GN=ATP2A2 PE=1 SV=1
Q9UDY2	ZO2_HUMAN	112.37	14	14	2.50E+06	21	19	24	Carbamidomethylation	Oxidation (M)	133958	Tight junction protein ZO-2 OS=Homo sapiens OX=9606 GN=TJP2 PE=1 SV=2
P08621	RU17_HUMAN	108.03	19	19	1.86E+06	9	9	13			51557	U1 small nuclear ribonucleoprotein 70 kDa OS=Homo sapiens OX=9606 GN=SNRNP70 PE=1 SV=2
Q9NR30	DDX21_HUMAN	104.2	18	18	2.01E+06	15	12	17			87344	Nucleolar RNA helicase 2 OS=Homo sapiens OX=9606 GN=DDX21 PE=1 SV=5
Q12797	ASPH_HUMAN	103.01	17	17	3.38E+06	14	14	18	Carbamidomethylation	Oxidation (M)	85863	Aspartyl/asparaginyl beta-hydroxylase OS=Homo sapiens OX=9606 GN=ASPH PE=1 SV=3
Q8N9T8	KRI1_HUMAN	101.85	15	15	2.40E+06	10	10	12			82598	Protein KRI1 homolog OS=Homo sapiens OX=9606

												GN=KRI1 PE=1 SV=3
Q15029	U5S1_HUMAN	97.72	9	9	2.36E+06	11	11	15			109436	116 kDa U5 small nuclear ribonucleoprotein component OS=Homo sapiens OX=9606 GN=EFTUD2 PE=1 SV=1
Q9NZB2	F120A_HUMAN	95.14	9	9	1.60E+06	11	10	11	Carbamidomethylation		121888	Constitutive coactivator of PPAR-gamma-like protein 1 OS=Homo sapiens OX=9606 GN=FAM120A PE=1 SV=2
O00267	SPT5H_HUMAN	94.27	9	9	1.87E+06	12	12	15	Oxidation (M)		121000	Transcription elongation factor SPT5 OS=Homo sapiens OX=9606 GN=SUPT5H PE=1 SV=1
Q9Y2W1	TR150_HUMAN	93.81	8	8	1.56E+06	9	9	11			108666	Thyroid hormone receptor-associated protein 3 OS=Homo sapiens OX=9606 GN=THRAP3 PE=1 SV=2
P11498	PYC_HUMAN	93.55	6	6	2.05E+05	7	7	8	Carbamidomethylation		129634	Pyruvate carboxylase mitochondrial OS=Homo sapiens OX=9606 GN=PC PE=1 SV=2
Q16531	DDB1_HUMAN	91.34	8	8	1.68E+06	10	10	12	Oxidation (M)		126968	DNA damage-binding protein 1 OS=Homo sapiens OX=9606 GN=DDB1 PE=1 SV=1
P02786	TFR1_HUMAN	90.81	12	12	1.09E+06	9	9	9			84871	Transferrin receptor protein 1 OS=Homo sapiens OX=9606 GN=TFRC PE=1 SV=2
P05067	A4_HUMAN	89.08	11	11	3.32E+06	10	10	16	Carbamidomethylation	Oxidation (M)	86943	Amyloid-beta precursor protein OS=Homo sapiens OX=9606 GN=APP PE=1 SV=3

Q16643	DREB_HUMAN	88.64	14	14	1.01E+06	9	9	10	Oxidation (M)	Acetylation (Protein N-term)	71429	Drebrin OS=Homo sapiens OX=9606 GN=DBN1 PE=1 SV=4
O00159	MYO1C_HUMAN	88.56	8	8	7.20E+05	9	9	9			121682	Unconventional myosin-Ic OS=Homo sapiens OX=9606 GN=MYO1C PE=1 SV=4
Q96A65	EXOC4_HUMAN	87.11	7	7	4.01E+05	6	6	8			110498	Exocyst complex component 4 OS=Homo sapiens OX=9606 GN=EXOC4 PE=1 SV=1
P28370	SMCA1_HUMAN	86.9	10	10	1.09E+06	13	13	13	Carbamidomethylation		122605	Probable global transcription activator SNF2L1 OS=Homo sapiens OX=9606 GN=SMARCA1 PE=1 SV=2
Q7L2J0	MEPCE_HUMAN	86.27	7	7	7.11E+05	6	6	7	Oxidation (M)		74355	7SK snRNA methylphosphate capping enzyme OS=Homo sapiens OX=9606 GN=MEPCE PE=1 SV=1
P27694	RFA1_HUMAN	85.76	16	16	1.09E+06	10	10	13			68138	Replication protein A 70 kDa DNA-binding subunit OS=Homo sapiens OX=9606 GN=RPA1 PE=1 SV=2
P35222	CTNB1_HUMAN	84.67	10	10	4.66E+05	9	8	11			85497	Catenin beta-1 OS=Homo sapiens OX=9606 GN=CTNNB1 PE=1 SV=1
Q9BUF5	TBB6_HUMAN	81.17	10	10	2.81E+04	4	1	6	Carbamidomethylation	Oxidation (M)	49857	Tubulin beta-6 chain OS=Homo sapiens OX=9606 GN=TUBB6 PE=1 SV=1
P81605	DCD_HUMAN	79.74	42	42	2.73E+06	6	6	7			11284	Dermcidin OS=Homo sapiens OX=9606 GN=DCD PE=1 SV=2
P36578	RL4_HUMAN	79.67	11	11	1.48E+06	5	5	6	Carbamidomet	Oxidation (M)	47697	60S ribosomal protein L4 OS=Homo sapiens

									hylation		OX=9606 GN=RPL4 PE=1 SV=5
O00469	PLOD2_HUMAN	79.34	7	7	2.95E+05	5	5	6		84686	Procollagen-lysine 2-oxoglutarate 5-dioxygenase 2 OS=Homo sapiens OX=9606 GN=PLOD2 PE=1 SV=2
O00203	AP3B1_HUMAN	78.76	4	4	2.74E+05	6	6	6		121320	AP-3 complex subunit beta-1 OS=Homo sapiens OX=9606 GN=AP3B1 PE=1 SV=3
Q8NFZ0	FBH1_HUMAN	77.78	7	7	3.63E+05	7	7	11		117686	F-box DNA helicase 1 OS=Homo sapiens OX=9606 GN=FBH1 PE=1 SV=2
P35606	COPB2_HUMAN	76.14	8	8	4.72E+05	9	9	10		102487	Coatomer subunit beta' OS=Homo sapiens OX=9606 GN=COPB2 PE=1 SV=2
P21127	CD11B_HUMAN	75.5	11	11	1.56E+06	10	10	11	Carbamidomethylation	92620	Cyclin-dependent kinase 11B OS=Homo sapiens OX=9606 GN=CDK11B PE=1 SV=4
Q9UQ88	CD11A_HUMAN	75.5	10	10	1.50E+06	9	9	10	Carbamidomethylation	91362	Cyclin-dependent kinase 11A OS=Homo sapiens OX=9606 GN=CDK11A PE=1 SV=4
Q9UBV2	SE1L1_HUMAN	75.29	3	3	4.58E+05	3	3	4		88755	Protein sel-1 homolog 1 OS=Homo sapiens OX=9606 GN=SEL1L PE=1 SV=3
Q13085	ACACA_HUMAN	74.16	4	4	8.51E+05	10	10	11		265551	Acetyl-CoA carboxylase 1 OS=Homo sapiens OX=9606 GN=ACACA PE=1 SV=2
Q9Y520	PRC2C_HUMAN	74	2	2	7.61E+05	5	5	6		316911	Protein PRRC2C OS=Homo sapiens OX=9606 GN=PRRC2C PE=1 SV=4

Q9P0K7	RAI14_HUMAN	73.99	8	8	2.16E+05	8	8	8		110041	Ankycorbin OS=Homo sapiens OX=9606 GN=RAI14 PE=1 SV=2
P08670	VIME_HUMAN	73.54	10	10	3.13E+05	5	4	7		53652	Vimentin OS=Homo sapiens OX=9606 GN=VIM PE=1 SV=4
P46977	STT3A_HUMAN	73.53	4	4	1.39E+05	3	3	3		80530	Dolichyl-diphosphooligosaccharide--protein glycosyltransferase subunit STT3A OS=Homo sapiens OX=9606 GN=STT3A PE=1 SV=2
Q8TDD1	DDX54_HUMAN	72.52	4	4	1.47E+05	4	4	4		98595	ATP-dependent RNA helicase DDX54 OS=Homo sapiens OX=9606 GN=DDX54 PE=1 SV=2
P42224	STAT1_HUMAN	70.85	5	5	2.20E+05	4	4	5		87335	Signal transducer and activator of transcription 1-alpha/beta OS=Homo sapiens OX=9606 GN=STAT1 PE=1 SV=2
Q6PKG0	LARP1_HUMAN	70.5	5	5	2.85E+05	6	6	6		123510	La-related protein 1 OS=Homo sapiens OX=9606 GN=LARP1 PE=1 SV=2
P61247	RS3A_HUMAN	70.47	19	19	7.17E+05	6	6	6		29945	40S ribosomal protein S3a OS=Homo sapiens OX=9606 GN=RPS3A PE=1 SV=2
Q5SSJ5	HP1B3_HUMAN	70.36	10	10	4.97E+05	6	6	7		61207	Heterochromatin protein 1-binding protein 3 OS=Homo sapiens OX=9606 GN=HP1BP3 PE=1 SV=1
P46777	RL5_HUMAN	67.27	12	12	1.06E+06	6	6	6		34363	60S ribosomal protein L5 OS=Homo sapiens OX=9606 GN=RPL5 PE=1 SV=3

O95490	AGRL2_HUMAN	65.22	5	5	1.29E+05	6	6	6	Carbamidomethylation	163348	Adhesion G protein-coupled receptor L2 OS=Homo sapiens OX=9606 GN=ADGRL2 PE=1 SV=2
Q9NYF8	BCLF1_HUMAN	64.87	6	6	6.34E+05	6	6	6		106122	Bcl-2-associated transcription factor 1 OS=Homo sapiens OX=9606 GN=BCLAF1 PE=1 SV=2
Q9BPX3	CND3_HUMAN	64.74	3	3	7.91E+05	4	4	4	Carbamidomethylation	114334	Condensin complex subunit 3 OS=Homo sapiens OX=9606 GN=NCAPG PE=1 SV=1

Table S2. Proteins specifically bound to biotin-labeled bi-(AID-1-T)

Protein ID	Accession	-10lgP	Coverage (%)	Coverage (%) Sample 1	Area Sample 1	#Peptides	#Unique	#Spec Sample 1	PTM		Avg. Mass	Description
P08621-2	RU17_HUMAN	146.32	25	25	6.95E+08	9	9	27	Oxidation (M)	Deamidation (NQ)	50618	Isoform 2 of U1 small nuclear ribonucleoprotein 70 kDa OS=Homo sapiens OX=9606 GN=SNRNP70
P08621	RU17_HUMAN	146.32	24	24	6.95E+08	9	9	27	Oxidation (M)	Deamidation (NQ)	51557	U1 small nuclear ribonucleoprotein 70 kDa OS=Homo sapiens OX=9606 GN=SNRNP70 PE=1 SV=2
O76021	RL1D1_HUMAN	92.3	11	11	6.86E+07	5	5	6	Carbamidomethylation		54973	Ribosomal L1 domain-containing protein 1 OS=Homo sapiens OX=9606 GN=RSL1D1 PE=1 SV=3

O60506-2	HNRPQ_HUMAN	66.87	4	4	1.15E+07	3	3	3		65682	Isoform 2 of Heterogeneous nuclear ribonucleoprotein Q OS=Homo sapiens OX=9606 GN=SYNCRIP
O60506	HNRPQ_HUMAN	66.87	4	4	1.15E+07	3	3	3		69603	Heterogeneous nuclear ribonucleoprotein Q OS=Homo sapiens OX=9606 GN=SYNCRIP PE=1 SV=2
O43390-4	HNRPR_HUMAN	66.87	4	4	1.15E+07	3	3	3		59953	Isoform 4 of Heterogeneous nuclear ribonucleoprotein R OS=Homo sapiens OX=9606 GN=HNRNPR
O43390	HNRPR_HUMAN	66.87	4	4	1.15E+07	3	3	3		70943	Heterogeneous nuclear ribonucleoprotein R OS=Homo sapiens OX=9606 GN=HNRNPR PE=1 SV=1
O43390-2	HNRPR_HUMAN	66.87	4	4	1.15E+07	3	3	3		71214	Isoform 2 of Heterogeneous nuclear ribonucleoprotein R OS=Homo sapiens OX=9606 GN=HNRNPR
P27694	RFA1_HUMAN	62.93	6	6	5.18E+06	3	3	5		68138	Replication protein A 70 kDa DNA-binding subunit OS=Homo sapiens

											OX=9606 GN=RPA1 PE=1 SV=2
Q8IYB3-2	SRRM1_HUMAN	62.27	4	4	6.64E+06	3	3	5	Oxidation (M)	102126	Isoform 2 of Serine/arginine repetitive matrix protein 1 OS=Homo sapiens OX=9606 GN=SRRM1
Q8IYB3	SRRM1_HUMAN	62.27	4	4	6.64E+06	3	3	5	Oxidation (M)	102335	Serine/arginine repetitive matrix protein 1 OS=Homo sapiens OX=9606 GN=SRRM1 PE=1 SV=2
Q9UNF1-2	MAGD2_HUMAN	61.47	7	7	1.14E+06	3	3	4	Deamidation (NQ)	63167	Isoform 2 of Melanoma-associated antigen D2 OS=Homo sapiens OX=9606 GN=MAGED2
Q9UNF1	MAGD2_HUMAN	61.47	7	7	1.14E+06	3	3	4	Deamidation (NQ)	64954	Melanoma-associated antigen D2 OS=Homo sapiens OX=9606 GN=MAGED2 PE=1 SV=2
P26368-2	U2AF2_HUMAN	60.81	5	5	1.68E+07	3	3	5	Oxidation (M)	53121	Isoform 2 of Splicing factor U2AF 65 kDa subunit OS=Homo sapiens OX=9606 GN=U2AF2
P26368	U2AF2_HUMAN	60.81	5	5	1.68E+07	3	3	5	Oxidation (M)	53501	Splicing factor U2AF 65 kDa subunit OS=Homo sapiens OX=9606

												GN=U2AF2 PE=1 SV=4
Q12797-7	ASPH_HUMAN	54.46	12	12	9.26E+05	1	1	3	Deamidation (NQ)	21963	Isoform 7 of Aspartyl/asparaginyl beta-hydroxylase OS=Homo sapiens OX=9606 GN=ASPH
Q12797-6	ASPH_HUMAN	54.46	9	9	9.26E+05	1	1	3	Deamidation (NQ)	29757	Isoform 6 of Aspartyl/asparaginyl beta-hydroxylase OS=Homo sapiens OX=9606 GN=ASPH
Q12797-11	ASPH_HUMAN	54.46	9	9	9.26E+05	1	1	3	Deamidation (NQ)	32420	Isoform 11 of Aspartyl/asparaginyl beta-hydroxylase OS=Homo sapiens OX=9606 GN=ASPH
Q12797-2	ASPH_HUMAN	54.46	8	8	9.26E+05	1	1	3	Deamidation (NQ)	34646	Isoform 2 of Aspartyl/asparaginyl beta-hydroxylase OS=Homo sapiens OX=9606 GN=ASPH
Q12797	ASPH_HUMAN	54.46	3	3	9.26E+05	1	1	3	Deamidation (NQ)	85863	Aspartyl/asparaginyl beta-hydroxylase OS=Homo sapiens OX=9606 GN=ASPH PE=1 SV=3
Q9Y2X3	NOP58_HUMAN	54.24	3	3	4.50E+06	1	1	1	Carbamidomethylation	59578	Nucleolar protein 58 OS=Homo sapiens OX=9606 GN=NOP58 PE=1 SV=1

Q15428	SF3A2_HUMAN	47.72	3	3	1.68E+06	1	1	4	49256	Splicing factor 3A subunit 2 OS=Homo sapiens OX=9606 GN=SF3A2 PE=1 SV=2
Q9UQ35	SRRM2_HUMAN	46.67	1	1	0	2	2	2	299616	Serine/arginine repetitive matrix protein 2 OS=Homo sapiens OX=9606 GN=SRRM2 PE=1 SV=2
P0CG39	POTEJ_HUMAN	41.25	1	1	4.66E+06	1	1	1	117390	POTE ankyrin domain family member J OS=Homo sapiens OX=9606 GN=POTEJ PE=3 SV=1
P63267-2	ACTH_HUMAN	41.25	3	3	4.66E+06	1	1	1	37083	Isoform 2 of Actin gamma-enteric smooth muscle OS=Homo sapiens OX=9606 GN=ACTG2
P63267	ACTH_HUMAN	41.25	3	3	4.66E+06	1	1	1	41877	Actin gamma-enteric smooth muscle OS=Homo sapiens OX=9606 GN=ACTG2 PE=1 SV=1
P62736	ACTA_HUMAN	41.25	3	3	4.66E+06	1	1	1	42009	Actin aortic smooth muscle OS=Homo sapiens OX=9606 GN=ACTA2 PE=1 SV=1

P68032	ACTC_HUMAN	41.25	3	3	4.66E+06	1	1	1		42019	Actin alpha cardiac muscle 1 OS=Homo sapiens OX=9606 GN=ACTC1 PE=1 SV=1
P68133	ACTS_HUMAN	41.25	3	3	4.66E+06	1	1	1		42051	Actin alpha skeletal muscle OS=Homo sapiens OX=9606 GN=ACTA1 PE=1 SV=1
A5A3E0	POTEF_HUMAN	41.25	1	1	4.66E+06	1	1	1		121444	POTE ankyrin domain family member F OS=Homo sapiens OX=9606 GN=POTEF PE=1 SV=2
Q6S8J3	POTEE_HUMAN	41.25	1	1	4.66E+06	1	1	1		121363	POTE ankyrin domain family member E OS=Homo sapiens OX=9606 GN=POTEE PE=2 SV=3
P0CG38	POTEI_HUMAN	41.25	1	1	4.66E+06	1	1	1		121282	POTE ankyrin domain family member I OS=Homo sapiens OX=9606 GN=POTEI PE=3 SV=1
Q08170	SRSF4_HUMAN	39.77	4	4	5.46E+05	2	2	2		56678	Serine/arginine-rich splicing factor 4 OS=Homo sapiens OX=9606 GN=SRSF4 PE=1 SV=2

Q9Y6M1-5	IF2B2_HUMAN	38.34	2	2	0	1	1	3		54722	Isoform 5 of Insulin-like growth factor 2 mRNA-binding protein 2 OS=Homo sapiens OX=9606 GN=IGF2BP2
Q9Y6M1-6	IF2B2_HUMAN	38.34	2	2	0	1	1	3		58578	Isoform 6 of Insulin-like growth factor 2 mRNA-binding protein 2 OS=Homo sapiens OX=9606 GN=IGF2BP2
Q9Y6M1-3	IF2B2_HUMAN	38.34	2	2	0	1	1	3		59001	Isoform 3 of Insulin-like growth factor 2 mRNA-binding protein 2 OS=Homo sapiens OX=9606 GN=IGF2BP2
Q9Y6M1-4	IF2B2_HUMAN	38.34	2	2	0	1	1	3		59666	Isoform 4 of Insulin-like growth factor 2 mRNA-binding protein 2 OS=Homo sapiens OX=9606 GN=IGF2BP2
Q9Y6M1-1	IF2B2_HUMAN	38.34	2	2	0	1	1	3		61843	Isoform 2 of Insulin-like growth factor 2 mRNA-binding protein 2

											OS=Homo sapiens OX=9606 GN=IGF2BP2
Q9Y6M1	IF2B2_HUMAN	38.34	2	2	0	1	1	3		66121	Insulin-like growth factor 2 mRNA-binding protein 2 OS=Homo sapiens OX=9606 GN=IGF2BP2 PE=1 SV=2
Q02040	AK17A_HUMAN	37.73	3	3	1.84E+05	2	2	2	Carbamidomethylation	80736	A-kinase anchor protein 17A OS=Homo sapiens OX=9606 GN=AKAP17A PE=1 SV=2
O76094-2	SRP72_HUMAN	34.81	3	3	1.73E+06	2	2	2		67880	Isoform 2 of Signal recognition particle subunit SRP72 OS=Homo sapiens OX=9606 GN=SRP72
O76094	SRP72_HUMAN	34.81	2	2	1.73E+06	2	2	2		74606	Signal recognition particle subunit SRP72 OS=Homo sapiens OX=9606 GN=SRP72 PE=1 SV=3
P05089-3	ARGI1_HUMAN	31.36	3	3	2.11E+05	1	1	1		25356	Isoform 3 of Arginase-1 OS=Homo sapiens OX=9606 GN=ARG1
P05089	ARGI1_HUMAN	31.36	2	2	2.11E+05	1	1	1		34735	Arginase-1 OS=Homo sapiens OX=9606

											GN=ARG1 PE=1 SV=2
P05089-2	ARGI1_HUMAN	31.36	2	2	2.11E+05	1	1	1		35664	Isoform 2 of Arginase-1 OS=Homo sapiens OX=9606 GN=ARG1
P27797	CALR_HUMAN	30.99	2	2	5.69E+06	1	1	1		48142	Calreticulin OS=Homo sapiens OX=9606 GN=CALR PE=1 SV=1
Q96PZ2	F111A_HUMAN	30.99	1	1	5.92E+04	1	1	1		70196	Serine protease FAM111A OS=Homo sapiens OX=9606 GN=FAM111A PE=1 SV=2
O00425-2	IF2B3_HUMAN	29.58	5	5	2.11E+05	1	1	2		21630	Isoform 2 of Insulin-like growth factor 2 mRNA-binding protein 3 OS=Homo sapiens OX=9606 GN=IGF2BP3
O00425	IF2B3_HUMAN	29.58	2	2	2.11E+05	1	1	2		63705	Insulin-like growth factor 2 mRNA-binding protein 3 OS=Homo sapiens OX=9606 GN=IGF2BP3 PE=1 SV=2
Q9UHX1-4	PUF60_HUMAN	27.48	2	2	3.97E+06	1	1	1	Oxidation (M)	54025	Isoform 4 of Poly(U)-binding-splicing factor PUF60 OS=Homo sapiens OX=9606 GN=PUF60

Q9UHX1-6	PUF60_HUMAN	27.48	2	2	3.97E+06	1	1	1	Oxidation (M)	55400	Isoform 6 of Poly(U)-binding-splicing factor PUF60 OS=Homo sapiens OX=9606 GN=PUF60
Q9UHX1-3	PUF60_HUMAN	27.48	2	2	3.97E+06	1	1	1	Oxidation (M)	55729	Isoform 3 of Poly(U)-binding-splicing factor PUF60 OS=Homo sapiens OX=9606 GN=PUF60
Q9UHX1-5	PUF60_HUMAN	27.48	2	2	3.97E+06	1	1	1	Oxidation (M)	57104	Isoform 5 of Poly(U)-binding-splicing factor PUF60 OS=Homo sapiens OX=9606 GN=PUF60
Q9UHX1-2	PUF60_HUMAN	27.48	1	1	3.97E+06	1	1	1	Oxidation (M)	58172	Isoform 2 of Poly(U)-binding-splicing factor PUF60 OS=Homo sapiens OX=9606 GN=PUF60
Q9UHX1	PUF60_HUMAN	27.48	1	1	3.97E+06	1	1	1	Oxidation (M)	59876	Poly(U)-binding-splicing factor PUF60 OS=Homo sapiens OX=9606 GN=PUF60 PE=1 SV=1
Q5VWX1	KHDR2_HUMAN	27.42	2	2	1.98E+05	1	1	1		38927	KH domain-containing RNA-binding signal transduction-associated protein 2 OS=Homo sapiens

										OX=9606 GN=KHDRBS2 PE=1 SV=1
Q07666-2	KHDR1_HUMAN	27.42	2	2	1.98E+05	1	1	1	45861	Isoform 2 of KH domain-containing RNA-binding signal transduction-associated protein 1 OS=Homo sapiens OX=9606 GN=KHDRBS1
Q07666	KHDR1_HUMAN	27.42	2	2	1.98E+05	1	1	1	48227	KH domain-containing RNA-binding signal transduction-associated protein 1 OS=Homo sapiens OX=9606 GN=KHDRBS1 PE=1 SV=1
Q69YN2-3	C19L1_HUMAN	26.65	2	2	6.65E+04	1	1	1	45537	Isoform 3 of CWF19-like protein 1 OS=Homo sapiens OX=9606 GN=CWF19L1
Q69YN2	C19L1_HUMAN	26.65	2	2	6.65E+04	1	1	1	60619	CWF19-like protein 1 OS=Homo sapiens OX=9606 GN=CWF19L1 PE=1 SV=2
P07910-4	HNRPC_HUMAN	26.24	4	4	5.68E+05	1	1	1	27821	Isoform 4 of Heterogeneous nuclear ribonucleoproteins C1/C2 OS=Homo sapiens

										OX=9606 GN=HNRNPC
P07910-2	HNRPC_HUMAN	26.24	3	3	5.68E+05	1	1	1	32338	Isoform C1 of Heterogeneous nuclear ribonucleoproteins C1/C2 OS=Homo sapiens OX=9606 GN=HNRNPC
P07910	HNRPC_HUMAN	26.24	3	3	5.68E+05	1	1	1	33670	Heterogeneous nuclear ribonucleoproteins C1/C2 OS=Homo sapiens OX=9606 GN=HNRNPC PE=1 SV=4
P07910-3	HNRPC_HUMAN	26.24	4	4	5.68E+05	1	1	1	25230	Isoform 3 of Heterogeneous nuclear ribonucleoproteins C1/C2 OS=Homo sapiens OX=9606 GN=HNRNPC
P12956-2	XRCC6_HUMAN	25.66	2	2	1.79E+05	1	1	1	65149	Isoform 2 of X-ray repair cross-complementing protein 6 OS=Homo sapiens OX=9606 GN=XRCC6
P12956	XRCC6_HUMAN	25.66	1	1	1.79E+05	1	1	1	69843	X-ray repair cross-complementing protein 6 OS=Homo sapiens OX=9606 GN=XRCC6 PE=1 SV=2
Q9NYF8-3	BCLF1_HUMAN	24.02	1	1	8.03E+04	1	1	1	100232	Isoform 3 of Bcl-2-associated transcription

									factor 1 OS=Homo sapiens OX=9606 GN=BCLAF1
Q9NYF8-2	BCLF1_HUMAN	24.02	1	1	8.03E+04	1	1	1	105948	Isoform 2 of Bcl-2-associated transcription factor 1 OS=Homo sapiens OX=9606 GN=BCLAF1
Q9NYF8	BCLF1_HUMAN	24.02	1	1	8.03E+04	1	1	1	106122	Bcl-2-associated transcription factor 1 OS=Homo sapiens OX=9606 GN=BCLAF1 PE=1 SV=2
P61978	HNRPK_HUMAN	23.81	2	2	1.03E+05	1	1	1	50976	Heterogeneous nuclear ribonucleoprotein K OS=Homo sapiens OX=9606 GN=HNRNPK PE=1 SV=1
P61978-2	HNRPK_HUMAN	23.81	2	2	1.03E+05	1	1	1	51028	Isoform 2 of Heterogeneous nuclear ribonucleoprotein K OS=Homo sapiens OX=9606 GN=HNRNPK
Q96I24	FUBP3_HUMAN	23.32	2	2	3.53E+05	1	1	1	61640	Far upstream element-binding protein 3 OS=Homo sapiens OX=9606 GN=FUBP3 PE=1 SV=2
Q86YZ3	HORN_HUMAN	23.21	0	0	6.11E+05	1	1	1	282389	Hornerin OS=Homo sapiens

											OX=9606 GN=HRNR PE=1 SV=2
Q9H2U1-3	DHX36_HUMAN	23.15	1	1	1.13E+06	1	1	1		111479	Isoform 3 of ATP-dependent DNA/RNA helicase DHX36 OS=Homo sapiens OX=9606 GN=DHX36
Q9H2U1-2	DHX36_HUMAN	23.15	1	1	1.13E+06	1	1	1		113153	Isoform 2 of ATP-dependent DNA/RNA helicase DHX36 OS=Homo sapiens OX=9606 GN=DHX36
Q9H2U1	DHX36_HUMAN	23.15	1	1	1.13E+06	1	1	1		114760	ATP-dependent DNA/RNA helicase DHX36 OS=Homo sapiens OX=9606 GN=DHX36 PE=1 SV=2
